# Ecotoxicity and Essential Properties of Fine-Recycled Aggregate

**DOI:** 10.3390/ma14020463

**Published:** 2021-01-19

**Authors:** Diana Mariaková, Klára Anna Mocová, Kristina Fořtová, Pavla Ryparová, Jan Pešta, Tereza Pavlů

**Affiliations:** 1 Research Team Architecture and the Environment, University Centre for Energy Efficient Buildings of Technical University in Prague, Trinecka 1024, 273 43 Bustehrad, Czech Republic; kristina.fortova@cvut.cz (K.F.); jan.pesta@cvut.cz (J.P.); tereza.pavlu@cvut.cz (T.P.); 2 Department of Environmental Chemistry, Faculty of Environmental Technology of University of Chemistry and Technology, Technicka 5, 166 28 Prague, Czech Republic; klara.bajerova@vscht.cz; 3 Department of Building Structures, Faculty of Civil Engineering of Technical University in Prague, Thakurova 7, 160 00 Prague, Czech Republic; pavla.ryparova@fsv.cvut.cz

**Keywords:** chemical properties, recycled concrete aggregate, ecotoxicity

## Abstract

This article deals with the possibility of utilization of secondary-raw materials as a natural sand replacement in concrete. Four types of waste construction materials were examined—recycled aggregate from four different sources. The natural aggregate was examined as well as used as the reference sample. All the samples were tested to evaluate the water absorption, particle size distribution, and particle density. The basic chemical reactions in the view of ecotoxicology are investigated and measured based on Czech standards. Chemical analysis, *Lemna* growth inhibition test, freshwater algae, daphnia acute, and mustard germination toxicity test were made and discussed in this paper. Based on the physical and geometrical properties and ecotoxicology of examined waste materials, this work evaluated them as suitable for utilization in concrete as a sand replacement.

## 1. Introduction

Tendencies toward the use of secondary raw materials have emerged in recent years, given the fact that the most used building materials are completely dependent in the production on primary materials. These sources are decreasing and due to their non-renewable disposition, the use of secondary raw materials is a logical step. However, when replacing, it is always necessary to consider the material being worked with and, with regard to the required properties, to select replaceable components and secondary materials that could be used. Nevertheless, the summarization of the material properties is only the second step, first it is necessary to take into account the properties of the secondary raw materials themselves.

Many studies describe the mechanical, physical, and durability properties of recycled aggregate (RA) originated from construction and demolition waste (CDW). The higher content of impurities and also mortar complicates the use of the fine fraction RA as a partial or full replacement of natural sand. For the coarse fraction, this problem is reduced [1]. This fact is one of the main reasons that keep the standard description of the use of recycled material as a substitute for sand in concrete. However, it is necessary to define a standard for the use of waste materials because of the fact that the extraction of sea sand has a negative impact on the environment—erosion by the sea is affected as well as the behavior of waves. Local ecosystems are generally negatively affected by sand mining [2].

However, most studies do not pay sufficient attention to the properties of waste raw materials themselves. The results of experiments on the waste may affect its overall utilization. According to the standards, it is possible to use recycled concrete aggregate (RCA) with a content of concrete particles above 70% as a partial replacement for gross natural aggregate (NA). However, this possibility is limited by the type of application [3,4]. Nevertheless, the utilization of another CDW types utilization is not defined in Czech standards yet (recycle masonry aggregate (RMA) or fine RA).

Previous research has verified the higher water absorption and lower density of RA. According to the previous studies, the water absorption of fine RCA ranges between 4.3% and 13.1% and the dry density of fine RCA ranges from 1900 to 2360 kg/m^3^ [5]. The higher water absorption and lower density of RCA is caused by old mortar attached to the surface of original aggregate which is more porous and less dense than the aggregate particles [1]. This leads to a higher water absorption of RCA, which influences the effective water–cement ratio and has negative impact on the workability of the concrete mix. The main differences between RMA and NA are water absorption and particle density. The particle density of coarse RMA ranges between 2000 and 2500 kg/m^3^ and ranges from 1800 to 2700 kg/m^3^ for fine RMA [6,7,8,9], while the water absorption is several times higher (about 12–15% in various studies), which is about ten times higher than the water absorption of NA [6,10,11]. The higher water absorption of RMA is caused by more porous materials contained in this aggregate such as red bricks, aerated concrete, plasters, mortars etc.

Ecotoxicity is one of the indicators that can show the extent to which living organisms, or the entire ecosystem, can be affected [12]. Ecotoxicology is a science dealing with the impact of harmful substances on the environment. In particular, the toxic effect on organisms that must meet the given conditions during laboratory testing is investigated. It is necessary to deal with these properties, because from the point of view of the concept of circular economy, it is desirable to use the produced waste and re-involve it in the process. The evaluation of toxic effects can be performed by several methods, the evaluated criteria include, for example, mortality or the number of inhibited individuals. If it is not possible to evaluate these factors, other output data can be used, which are based on specific physiological characteristic of the organisms, such as growth of an individual or test colony or population can be considered as suitable parameters [13].

Such great importance for the basic properties of selected waste raw materials is placed mainly due to their use in concrete. The aim of the research is to analyze the properties of waste materials from an environmental point of view, because in materials such as concrete, it is now desirable to use secondary raw materials that reduce the negative impact on the environment. The production of concrete itself is a great burden for the environment, because of the need to quarry of primary raw materials, often to transport these raw materials and further processing. It is also necessary to consider the amount of concrete produced in the construction industry and subsequently the waste in this sector. Construction waste is often landfilled, which seems to be an inappropriate choice because of the potential for toxicity of some materials. Another possibility is the recycling of waste materials, a suitable choice here also seems to be the overall evaluation of the life cycle to confirm the suitability of this solution [14].

The concrete composition is still developing. Many possibilities to substitute usual components of concrete have been evaluated in the past few years, and the utilization of secondary raw materials such as RA, blast furnace slag, fly ash etc. The use of RA from CDW as a partial or full replacement of aggregate in concrete is solved in terms of mechanical properties in various researches [10,11,15,16,17]. This approach is necessary to evaluate the strength of new concretes containing waste, because of the following use. Over the past decades, the properties of RA and their influence on concrete characterization has been tested and evaluated. The research has become complex in the last years because the use of RA as a replacement for natural aggregate (NA) in concrete in one of the most effective approaches how to use CDW in the recycle process. Because of constantly increasing concrete production, the amount of CDW is growing as well [18].

The properties of recycled aggregate, especially its quality, are the main indicators when used in concrete. Because of the frequent occurrence of impurities, there is a risk of decreasing mechanical properties of the concrete mixture. Generally, the RCA has lower particle density and higher water absorption, which is caused by the porous mortar which is attached in the original aggregate surface and water absorption influences the workability of fresh concrete mixture [1,5]. It is assumed that although the fine particles have a larger surface area, their contents could fill the space between the larger particles of the aggregate, which can eliminate the porosity. When using the fine fraction of RCA, problems also arise in terms of physical properties. Because of the high content of cement mortar, higher water absorption has to be discussed and this phenomenon also occurs with the partial replacement of RCA compared to reference concrete. Increasing the w/c ratio results in better processability of concrete mixture [5].

A study [19] dealt with the possible use of fine RCA in concrete. The results reached a possible substitution rate of up to 30%, nevertheless, the laboratory-prepared fine RCA was used in this study, which could have affected the results. The difference between laboratory-prepared fine RCA and fine RCA from CDW can be negligible, although particle sizes of 125 to 500 µm show a high content of cement mortar, which could lead to better mechanical properties of the concrete [18]. So far, the results of the studies are more with a decreasing trend—the compressive strength decreases to 70% of the reference concrete mixture when replacing NA with fine RCA. In the case of partial replacement (25, 50, and 75%), the values of compressive strength in the study [6] were similar, but in the case of full replacement, the lowest compressive strength was measured on the concrete sample.

The properties of RMA may adversely affect the workability of fresh concrete or the mechanical properties of concrete [19]. The composition can also have an effect, as there is a risk of impurities content in the RMA. The high water absorption is the key property in the RMA utilization in concrete. There are two possibilities to compensate higher water absorption of RA and it is necessary to find the optimal solution for practice. The possibilities are: during the mixture designing, it is necessary to take into account the pre-soaking of the aggregate into water (24 h before mixing) [7], or count with the use of an additional water during mixing itself [8]. Different studies [6,7,8] examined different amount of NA replacement rates, most studies show a decrease in compressive strength after 28 days depending on the increasing RMA replacement rates.

For all the mixtures containing RMA, the measured compressive strength increased after 90 days. This increase was higher compared to reference concrete sample containing NA. The reason for this phenomenon is given by, for example, the formation of pozzolanic reactions due to the presence of silica and alumina contents in the bricks used or a lower w/c ratio of concrete mixtures containing fine RMA [6]. This assumption was demonstrated by further studies where the w/c ratio was calculated from RMA water absorption. The results of these studies do not show any significant changes in the rate of strength development between the age of 28 and 90 days [7,8].

This article is proceeding the utilization of waste materials—in particular, the CDW. The research is focused on three types of RCA and one type of RMA from different sources. The planned use of these materials is in concrete as a natural sand substitute. However, the properties of CDW materials may be inappropriate, it is necessary to experimentally verify at least the essential properties such as particle size distribution, chemical composition, water absorption, or particle density of the recycled waste materials used in comparison with the reference sample (natural sand). Among other things, it is necessary to think about the suitability of use due to the possible toxic effects on natural organisms, the possibility of releasing toxic substances, etc.

The usual utilization of fine RA as backfilling layers in the Czech Republic leads to the toxic pollution in the environment. In addition, the previous investigation of the research group showed the satisfying results of mechanical properties of concrete with almost full replacement of natural sand by fine RA [20]. The published results showed that the mechanical and deformation properties of mixtures with the content of FRA were similar (fine RMA) or slightly better (fine RCA) in comparison with the reference mixture.

This research work has responded to previous results and dealt with the possible utilization of this waste materials with prediction of the chosen waste materials properties, which will fulfill the requirements for use in concrete especially in the vie w of ecology to show the advantages of this solution. Environmental impacts occur mainly during the integration of the components of concrete with rain or wind. These processes can cause degradation, as various substances can leach out due to the action of water and it is desirable that the waste materials used in concrete do not have a toxic effect on the environment. Therefore, the chemical composition and possible toxic effects must be investigated.

There are a number of factors that can affect the overall effect, such as contact time, porosity, or material damage, which only a small number of studies have examined these days [12]. This study works with the new assumption that the use of waste materials in concrete leads to their immobilization. The relative topic is also dealt with in another study [21], which brings new insights into the potential use of RA in concrete.

## 2. Materials and Methodology

### 2.1. Materials

Within the performed experiments, waste materials from CDW were used. The NA, which is widely used in concrete, was tested as a reference. The RA that has been tested should have properties comparable to natural sand, as the aim is to replace these primary raw materials in concrete with the secondary raw materials mentioned below. The RA originated from a recycling centers in the Czech Republic and had different origins:

(1) RA 1 was prepared from reinforcement concrete in the recycling center by the two-steps recycling process. The crushed and separated recycled aggregate of fraction 16/128 mm from the first step of recycling process was crushed and sieved to the fractions in the second step.
(2) RA 2 originated from highway and was partially prepared in the recycling center to fraction 64/128 mm. Afterwards, the fraction 64/128 was crushed and sieved into the fractions in the laboratory.
(3) RA 3 originated from the ground floor structures and was partially prepared in the recycling center to fraction 64/128 mm. Afterwards, the fraction 64/128 was crushed and sieved into the fractions in the laboratory.
(4) RA 4 originated from the masonry structures and contains mostly red bricks, mortar, and plasters. It was prepared from reinforcement concrete in the recycling center by the two-steps recycling process. The crushed and separated recycled aggregate of fraction 16/128 mm from the first step of recycling process was crushed and sieved to the fractions in the second step.

Two types of RA (RA 1 and RA 4) were modified by the two-step recycling process. In the first step, the CDW is crushed and separated to three fractions 0/4, 4/16, and 16/128 mm; the fraction 0/4 and 4/16 mm is separated and used as downcycling material for instance as backfilling material due to the unwanted impurities such as soil and dust. In the second step, the fraction 16/128 mm was crushed again and sieved to the fraction 0/4, 4/8, and 8/16. The content of unwanted impurities is reduced by this process. The recycling process of two other RAs (RA 2 and RA 3) was similar, however, the RA of fraction 64/128 mm from the first step of recycling process was finally crushed and sieved in the laboratory to fractions 0/4, 4/8, and 8/16 mm. Although three fractions of RA were prepared, only fraction 0/4 mm was studied in this investigation.

**Figure 1:**
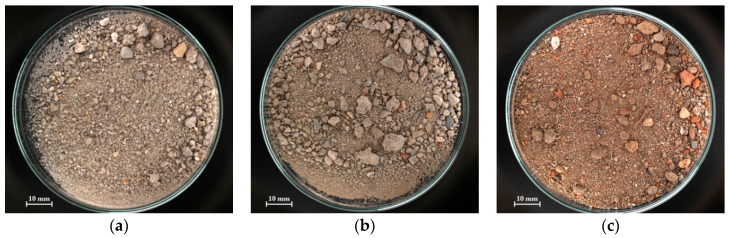
Tested waste materials recycled concrete aggregate (RCA): (**a**) NA; (**b**) RA 1; (**c**) RA 4.

All the samples were used as a fine aggregate and the experiments were performed according to the valid Czech standards. The used samples are shown in Figure 1.

### 2.2. Physical and Geometrical Properties

All types of aggregates were used in natural humidity conditions. However, for the particle size distribution test the aggregates were dried until stabilization of weight and further tested according to the CSN EN 933-1 [22]. Limits are defined in CSN EN 12620 [23]. Water absorption and density were verified by the pycnometric method according to CSN EN 1097 [24].

#### 2.2.1. Fineness Modulus

Fineness modulus (FM) is informative property of aggregate used for determination of the degree of uniformity of the aggregate gradation. It is an empirical number relating to the fineness of the aggregate. The higher the FM is, the coarser the aggregate is CSN EN 12620 + A1 [3]:

(1) $$\mathrm{FM}=\frac{\left[\left(X_{>4})+(X_{>2})+(X_{>1})+(X_{>0.5})+(X_{>0.25})+(X_{>0.125}\right)\right]}{100}$$

where *X* is the sample of an aggregate retained on each sieve [3].

Fineness modulus of aggregate primarily describes the coarseness and fineness of fine aggregate (CSN EN 12620 + A1, 2008 [3]).

#### 2.2.2. Fines Content

Determination of percentage of fines is based on the sieving test CSN EN 933-1 [22]. The requirements of the fines content in aggregate for concrete is listed in the Czech standard CSN EN 12620 + A1 [3]. According to this standard, the defined limit of maximal fines content in aggregate for concrete is 3% without further requirements.

Calculate the percentage of fines for dry method (*f*) passing the 0.063 mm sieve in accordance with the following Equation:

(2) $$f=\frac{P}{M}\;\times 100$$

where *f* is fines content (%), *M* is the mass of the test portion (kg), and *P* is the mass of the screened material remaining in the pan (kg) [25].

### 2.3. Ecotoxicity Experiments

Air-dried samples of 100 g were mixed with 1000 mL of H_2_O and homogenized on an overhead shaker (7 rpm) for 24 h [26]. Consequently, the solid particles in leachates were settled for 10 min and the liquid phase was centrifuged (2360× *g*, 10 min, 25 °C) and filtered through a membrane paper with pores of 4 μm. pH and electrical conductivity was determined in the filtrated leachates at room temperature. All the leachates were prepared in two replicates. Selected elements (Cu, Ni, Zn, As, Cd, Cr, Sb, Ba, Hg, Pb, Se, B, Mo, V, Ca, Na) were determined using atomic absorption spectrometry with flame atomizer 280FS AA developed by Agilent Technologies, Inc. (Santa Clara, CA, USA) in leachates after acidification by HCl to pH of 2.0.

Ecotoxicological bioassays were performed with both untreated leachates and leachates amended with relevant inorganic nutrients according to control media of the given test species. pH adjustment was not included in the leachate’s treatment.

#### 2.3.1. Freshwater Algae Toxicity Test

Algal growth inhibition test was performed with freshwater green algae *Desmodesmus subspicatus*, strain Brinkmann 1953/SAG 86.81 which was obtained from CCALA, IBOT, AS CR (Trebon, Czech Republic) partly following the ISO guideline 8692 [26]. Bold Basal Medium (BBM; pH 6.6 ± 0.2) according to [27] was used as the control medium. For the test 25 mL Erlenmeyer flasks were filled with 15 mL of leachate/control sample and inoculated with pre-cultivated algae (80 000 cells per 1 mL). Samples and controls were represented by triplicates or quadruplicates, respectively. Flasks were covered with sterile cellulose cap and placed under stable temperature (23 ± 1 °C), light cycle (16 h of light period; 6000–8000 l×), and continuous shaking (130 rpm) for 72 h. Algal cell density was determined via cell counting using microscope and Bürker chamber (Hecht, Germany).

For chlorophyll content determination 9 mL of algae in uspension was isolated by centrifugation (14,780× *g*, 10 min, 4 °C), 8 mL of pure methanol was added to sediment and homogenized. In case of algae clusters formation, dispersion of the cells was eased by placing into cooled ultrasonic bath for 2 min. After three days of extraction (dark, 4 °C, daily homogenization), extracts were centrifuged (14,780× *g*, 10 min, 4 °C) and absorbance at 653 and 666 nm was measured using spectrophotometer UV-1900 developed by Shimadzu Corporation (Kyoto, Japan). Total chlorophyll content per volume unit was calculated according to [28].

#### 2.3.2. Mustard Germination Toxicity Test

Germination test was performed with mustard (*Sinapis alba*), variety Severka C1. Seeds were obtained from Aros company (Prague, Czech Republic). Petri dishes (diameter of 9 cm) containing membrane paper soaked with 5 mL of medium/sample were prepared in triplicates (samples) or quadruplicates (controls). The control medium consisted of CaCl_2_·H_2_O~294 mg·L^−1^; MgSO_4_·7H_2_O~123.25 mg·L^−1^; NaHCO_3_~64.75 mg·L^−1^; and KCl~5.75 mg·L^−1^; pH of 7.8 ± 0.2 adjusted by 1 M HCl. 17 seeds of approximately 1.5 mm in diameter were placed in a regular net on the membrane paper. Covered dishes were stored under stable temperature (20 ± 1 °C) in dark for 72 h. After exposition, plant root length was determined.

#### 2.3.3. Lemna Growth Inhibition Test

Duckweed assay was proposed by ISO guideline 20079 [29] using *Lemna minor*, strain Steinberg originated from Federal Environmental Agency (Berlin, Germany). Steinberg medium modified by Altenburg (pH 5.5 ± 0.2) [29] served as the control. The test was carried out in 150 mL beaker filled with 100 mL of sample/control medium. Samples and controls were represented by three and five replicates, respectively. Each vessel was inoculated with 10 fronds of duckweed of a similar total frond area and covered with transparent film. Test vessels were kept in a stable temperature (24 ± 2 °C) and exposed to a light cycle (5000–6000 l×; 16 h light/8 h dark).

The total frond area was determined by image analysis using NIS Elements (Version 5.20, Laboratory Imaging, Prague, Czech Republic). Growth rate (GR) was calculated from the values based on repeated measurements during the test exposure, i.e., 0th, 3rd, and 7th day. After the 7-day exposition, fronds were extracted by pure methanol (48 h; 4 °C, dark) and the total chlorophyll content was determined spectrophotometrically (Shimadzu UV-1900, Shimadzu Corporation, Kyoto, Japan) according to [28].

#### 2.3.4. Daphnia Acute Toxicity Test

Acute toxicity assay was performed with *Daphnia magna* juveniles aged up to 24 h, which were hatched from ephippia obtained from Microbiotests Inc. (Mariakerke (Gent), Belgium). The experimental design was done following ISO guideline 6341 [30] with some adjustments. Fresh ADaM medium (pH~7.8 ± 0.2) prepared according to [31] was used as control sample.

Five juvenile individuals were transferred into 25 mL beakers with 20 mL of leachate or control sample, covered with transparent film and put under stable temperature (20 ± 1 °C) and light cycle (1000–2000 l×; 16 h light/8 h dark). Each sample was represented by four replicates, whereas controls by six replicates. The inhibition of daphnia mobility (viability) was observed after the 48 h exposition.

#### 2.3.5. Evaluation of Ecotoxicity Data

In algae and duckweed growth rate (GR) based on cell number and frond area respectively was calculated using Equation.

(3) $$r=\frac{\ln X_{t1}-\ln X_{t0}}{t_{1}-t_{0}}$$

where r is growth rate per day, *X_t_*_0_ is value of the parameter in *t*_0_ (d), and *Xt*_1_—value of the parameter in *t*_1_ (d). [29].

All the ecotoxicological data (algal and frond GR, root elongation, chlorophyll content and daphnia viability) were consequently expressed as the values of inhibition/stimulation in percentage, where tested organisms in leachates were compared to control organisms using the following Equation.

(4) $$I=\frac{X_{c0-\;}X_{ci}}{X_{c0}}\times 100$$

where *I* is inhibition/stimulation of growth (%), *X_c_*_0_ is average value of control, and *X_ci_*—average value of sample *i*. [29].

EC50 values were calculated from the inhibition data for all ecotoxicity tests using nonlinear regression.

## 3. Results and Discussion

### 3.1. Physical and Geometrical Properties

In the previous studies, it was found that the dry density of fine RCA (recycled concrete aggregate) ranges between 1900 and 2360 kg/m^3^ [5] and fine RMA (recycled masonry aggregate) between 2000 and 2500 kg/m^3^ [9,10,11,32,33,34] which is generally lower than natural sand. The range of water absorption of fine RCA ranges between 4.3% and 13.1% [5] and RMA from 12% to 15% [8,32,33], which is more than ten times higher than natural sand.

**Figure 2:**
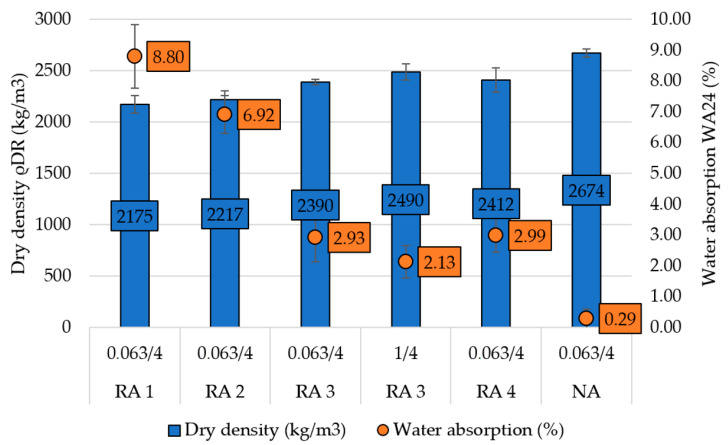
Oven-dry density and water absorption of aggregate according to CSN EN 1097-6.

In this study, granulometry, dry density and water absorption of one type of NA and four types of RA were examined and compared (Figure 2). All types of RA were prepared from construction and demolition waste and sieved into fraction of 0–4. All types of RCA contained more than 90% of recycled waste concrete (unbound natural aggregate and cement mortar) and RMA contained more than 70% of the waste masonry (red brick, aerated concrete, and plaster).

**Figure 3:**
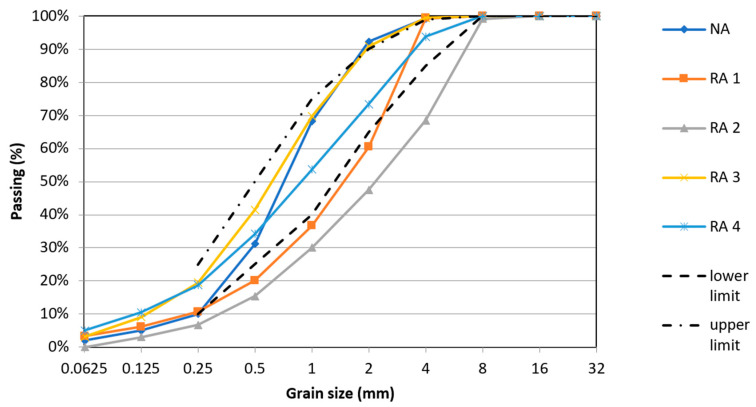
The particle size distributions of natural aggregate (NA) and RCA (grain size 0/4 mm) according to CSN EN 933-1.

All tested properties of RA differed from NA, especially the water absorption capacity, which was more than ten times higher and ranged from 2.1 to 8.8% for fine fraction of RA, where the highest water absorption was measured for RA 1 originated from waste concrete from recycling center. On the contrary the lowest water absorption was evaluated for RA 3, which originated from demolished floor structures and was partially prepared in the laboratory. The water absorption of RA 3 was measured for fraction 0.063/4 mm and 1/4 mm to show the influence of fines particles of aggregate. The results of water absorption show the slight relation with the fineness modulus. This evaluation shows slightly lower water absorption of RCA [5] and significantly lower water absorption of RMA [8,32,33] in comparison with the results of previous studies. The decline of dry density of fine RCA in comparison with NA ranges between 7 and 20% and the decline of dry density of fine RMA in comparison with NA is 10%. These results correspond with the results of previous studies [9,10,11,32,33,34]. The higher density was measured for RA3 of fraction 1/4 mm. Furthermore, the RA contains more fine particles and has different granulometry in comparison with NA and two examined types of aggregate (RA 1 and RA 2) does not meet the requirements in Standard [3] (see Figure 3). Therefore, the basic physical properties of aggregates (see Table 1) and the basic geometrical properties of fine aggregate (see Table 2) are presented to show the differences in the materials and its comparison with natural sand.

### 3.2. Ecotoxicity

Results showed different leaching behavior of the tested samples (Table 3 and Table 4). All of the leachates were alkaline; from weakly basic NA to strongly basic RA 2. Similarly, the electrical conductivity varied. Very low ions content was found in NA which reflected the inert character of this material. The conductivity values of RA 1 and RA 4 were relatively close to the values of the control media of test organisms whereas RA 2 and RA 3 showed very high levels. As Table 3 shows, the selected heavy metals were at very low concentrations or under the detection limit in all the samples. For instance, zinc which plays an important role as a microelement for photosynthesis, homeostasis, and growth of microalgae [35] was far below the Zn concentration in BBM medium [27]. In RA 2 leachate barium content (1.056 mg·L^−1^) was approximately 5–10 times higher in comparison with the other samples. However, concentrations below 5 mg·L^−1^ in freshwaters were found safe for duckweed [36] and acute toxicity for crustacean and green algae was observed in concentrations higher than 10 5 mg·L^−1^ [37] On the contrary the content of sodium and calcium was significant and showed the biggest differences among the leachates. The Ca content in RA 1 (80.08 5 mg·L^−1^) was equal or similar to the Ca content in Steinberg and ADaM media whereas in RA 2 (660.57 5 mg·L^−1^) the Ca content was 8–13 times higher.

**Figure 4:**
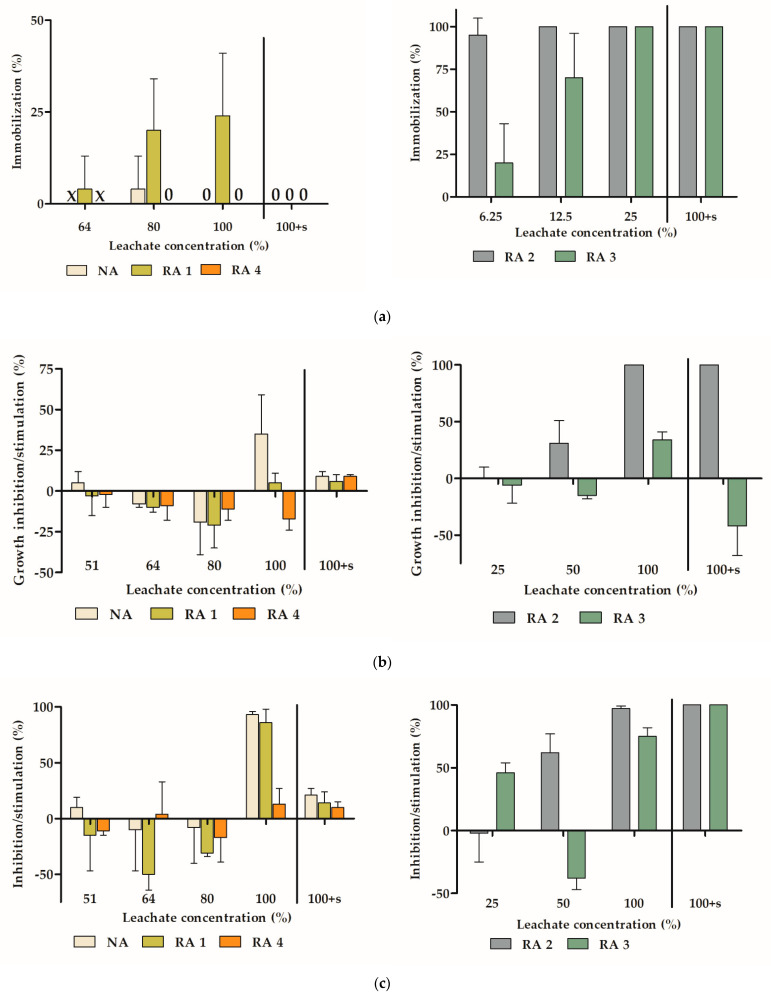

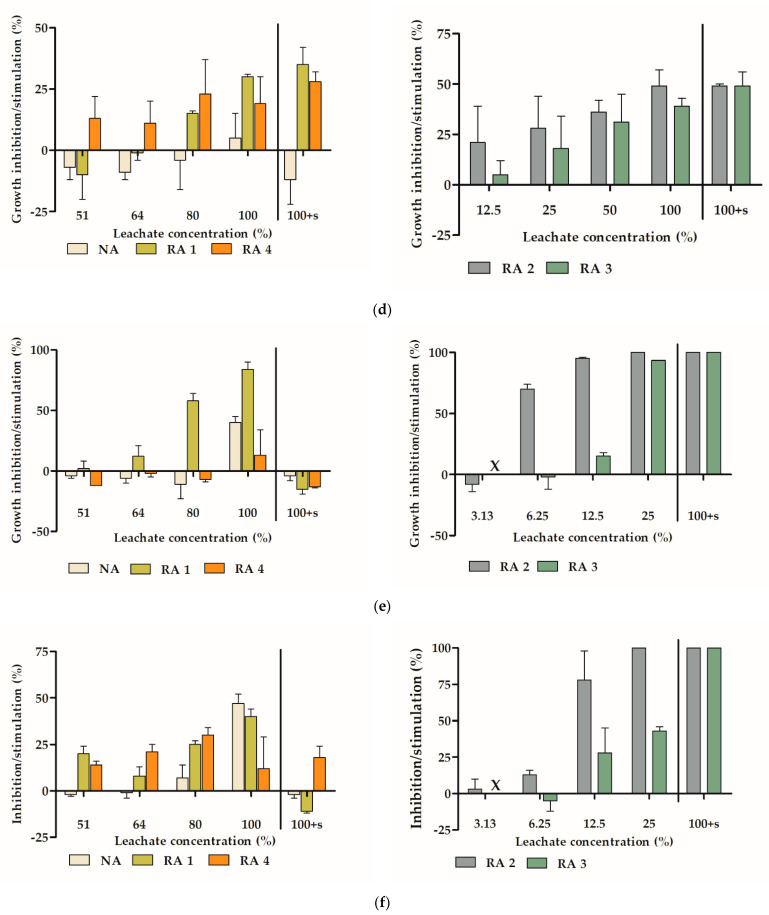
The results of ecotoxicity experiments: (**a**) Daphnia immobilization, (**b**) algae growth rate, (**c**) algae chlorophyll content, (**d**) mustard root elongation, (**e**) *Lemna* growth rate, (**f**) *Lemna* chlorophyll content. X—not determined, 0—zero values, 100 + s—leachates amended with nutrient salts.

**Figure 5:**
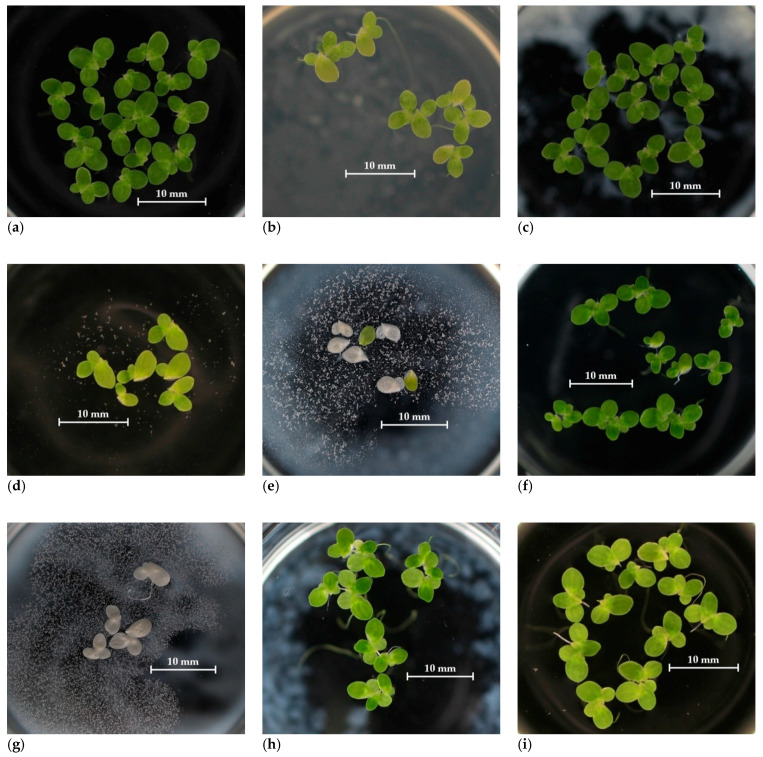
Test plants photo-documentation: (**a**) control, (**b**) NA—100%, (**c**) NA—salts, (**d**) RA 1—100%, (**e**) RA 2—12.5%, (**f**) RA 2—1.56%, (**g**) RA 3—100 + s, (**h**) RA 3—12.5 %, (**i**) RA 4—100%.

The response of the model organisms to the tested samples is shown in Figure 4. Generally, exposure to RA 4 leads to the lowest effect on test organisms with no effect on daphnia (Figure 4a) and only slight reduction of mustard root length (Figure 4c) and duckweed chlorophyll content (Figure 4f). The growth and chlorophyll reduction in both algae and duckweed (Figure 4b,e and Figure 5b,e,i) in non-treated 100% NA leachate was caused by the lack of minerals. However, there was no growth reduction after addition of nutrients or dilution of the non-treated leachates to 80%. In RA 1 leachate (non-treated and 80% dilution) growth inhibition effect was observed which was caused probably because of the higher pH of the sample (Table 3). Further dilution of the leachate led to pH decrease and no-effect or stimulation of the organisms’ growth. For the ecotoxicity comparison EC50 values were calculated for leachate concentration in % (Table 5). Classification of ecotoxicity level was done according to [38]. The calculated EC50 values of NA, RA 1, and RA 4 were higher than the concentrated leachates. Thus, according to [38] these samples were classified as non-toxic.

In RA 2 and RA 3 samples both untreated and nutrients-amended non-diluted leachates the lethal effect was observed for all the test organisms except for mustard (Figure 4 and Figure 5e,g). Because of higher toxicity effect, different dilution rates had to be performed in these leachates. Inhibitory or toxicity effect was shown mainly in duckweed growth, chlorophyll and especially daphnia. According to [38] these leachates were classified as inhibitory—mild toxic (Table 5). The ecotoxicity of concrete leachates can result both from toxic compounds presence and alkalinity [12,39,40]. Besides the high conductivity in RA 2 and particularly RA 3 (Table 4) the pH values remained highly alkaline even after dilution of the original leachates unlike in the case of NA, RA 1, and RA 4 samples. In accordance with our previous study [41] we hypothesize that the high conductivity might contribute to the buffering capacity of the leachate and thus maintain the toxicity of the samples with time and/or dilution. As a most probable reason of the alkalinity was the high content of calcium ions, however, this statement will have to be verified in the further testing.

The most sensitive bioindicator of ecotoxicity was daphnia which was already recommended for ecotoxicological assessment of concrete leachates [39,42]. Our study also confirmed duckweed as a suitable plant test organism because of its sensitivity where toxic effect can be detected on both morphological and biochemical level and various symptoms such as necrosis can be observed. Because of the photo-documentation of the test plants, the behavior of the sample within time can be also monitored, e.g., precipitation of the salts in leachates, especially in variants with nutrient addition or those with high conductivity values (Figure 5c).

Based on the ecotoxicity results, the samples RA 2 and RA 3 are suitable to verify the prediction that use of waste materials in concrete can lead to their immobilization. These samples were picked because of their properties, especially higher toxicity level (inhibitory—mild toxic), but other aspects should also be considered. RA 3 with lower water absorption and higher bulk-density also fulfills the limits of current standards in particle size distribution.

## 4. Conclusions

This research examined and discussed the experimental verification of physical and geometrical properties of recycled concrete aggregate from four different sources. Tested samples (RA 1–4) were measured and compared with the reference samples (NA). The ecotoxicity tests were made to verify the impact of harmful substances to environment. Based on provided experiments, the final conclusions are summarized in the following points:

- The water absorption of RA 1–4 is up to ten times higher than NA. The highest absorption was measured on RA 1, the lowest on RA 3.
- The highest density was measured on RA 3, which corresponds with the lowest water absorption measured on this sample.
- Samples RA 1 and RA 2 provided higher number of fine particles in the particle size distribution and the limits of the current standards have not been fulfilled.
- Ecotoxicity of the tested leachates increased from non-toxic effect in NA, RA 1, and RA 4 to inhibitory effect or mild toxicity in RA 2 and RA 3 in following order:

(5) NA ~ RA 1 ~ RA 4 < RA 3 ≤ RA 2

The novelty of this study is in the effort to create a comprehensive analysis of potential environmental threats in the ecotoxicology point of view considering the physical and geometrical properties of RA. This work presents the results of the effect of waste materials on leachates and ecotoxicity. Because of the higher mortality of the tested organisms, landfilling of these materials is not appropriate. A more suitable variant seems to be the use in concrete because of the new assumption that the use of waste materials in concrete leads to their immobilization. This assumption is verified in a follow-up research.

## Figures and Tables

**Table 1:** Basic geometrical properties.

Physical Properties	NA	RA 1	RA 2	RA 3	RA 4
	0/4 mm	0/4 mm	0/4 mm	0/4 mm	0/4 mm
Fineness modulus	2.10	2.90	2.57	1.92	2.53
σ	0.06	0.03	0.08	0.88	0.14
Content of fines	2.0%	3.4%	3.1%	3.2%	5.1%
σ	0.2%	0.0%	0.8%	1.7%	1.1%

**Table 2:** Basic physical properties.

Type of Aggregate	Fraction (mm)	Oven-Dry Density (kg/m^3^)		Water Absorption (%)	
		ρ _RD_	σ	WA_24_	σ
NA	0.063/4	2674	38	0.29	0.31
RA 1	0.063/4	2175	87	8.80	1.03
RA 2	0.063/4	2217	89	6.92	0.60
RA 3	0,063/4	2390	29	2.93	0.80
RA 3	1/4	2490	81	2.13	0.53
RA 4	0.063/4	2412	118	2.99	0.56

**Table 3:** Basic chemical properties.

Chemical Properties		NA	RA1	RA2	RA3	RA4
Leachates	pH	8.1 ± 0.6	10.3 ± 0.0	12.2 ± 0.0	11.7 ± 0.1	8.6 ± 0.0
	el. conductivity (µS·cm^−2^)	27 ± 3	545 ± 13	7150 ± 80	2730 ± 10	1129 ± 6
Element (mg/l)	Ca	3	80.08	660.57	146	216.98
	Na	1	13.733	8.83	9.92	20.71
	As	<0.4	<0.4	<0.3	<0.3	<0.4
	Zn	0.027	~0.012	~0.011	~0.008	0.023
	Cu	<0.01	~0.02	<0.02	<0.02	~0.02
	Cr	<0.05	~0.05	~0.1	<0.05	<0.05
	Ba	<0.1	<0.1	1.056	~0.2	<0.1
	Se	<0.4	<0.4	<0.5	<0.5	<0.4
	Pb	<0.04	<0.04	<0.06	<0.06	<0.04
	Hg	<0.001	<0.001	<0.001	<0.001	<0.001

**Table 4:** Properties of control media.

**Properties.** **of Control** **Media**	**pH**	***Lemna***	**Algae**	***Sinapis***	**Daphnia**
		5.5 ± 0.1	6.6 ± 0.1	7.8 ± 0.2	7.8 ± 0.2
	el. conductivity (µS·cm^−^^2^)	977 ± 15	853 ± 12	625 ± 7	829 ±15

**Table 5:** EC50 values and ecotoxicity assessment of leachates. GR—growth rate; chl—chlorophyll content; TC—toxicity class.

Type of Aggregate	Daphnia	Sinapis	Algae GR	Algae Chl	*Lemna* GR	*Lemna* Chl	Toxicity Level
NA							
EC50	˃100	˃100	~100	~97	~100	˃100	
TC	A1	A1	A2	A2	A1	A1	non-toxic
RA 1							
EC50	˃100	˃100	~100	~99	78	˃100	
TC	A2	A3	A2	A2	A3	A2	non-toxic
RA 2							
EC50	~5	˃100	~54	~49	~6	9	
TC	C	A3	A3	B	C	C	Inhibitory—mild toxic
RA 3							
EC50	~7	˃100	˃100	~95	16	26	
TC	C	A3	A1	B	B	B	Inhibitory—mild toxic
RA 4							
EC50	˃100	˃100	˃100	˃100	˃100	˃100	
TC	A1	A2	A2	A2	A1	A2	non-toxic

## Data Availability

Data available in a publicly accessible repository.
